# The protective efficacy of baricitinib against lipopolysaccharide / D-Galactosamine–Induced acute liver Injury

**DOI:** 10.3389/fphar.2026.1848559

**Published:** 2026-07-08

**Authors:** Khalid Khalaf Almutairi, Sara H. Hazem, Ghada M. Suddek

**Affiliations:** Department of Pharmacology and Toxicology, Faculty of Pharmacy, Mansoura University, Mansoura, Egypt

**Keywords:** acute liver failure, baricitinib, hepatoprotection, JAK-STAT pathway, NF-κB, oxidative stress

## Abstract

**Intorduction:**

Baricitinib (BARI), the selective JAK1/JAK2 inhibitor demonstrates enhanced immunological activity against several inflammatory conditions. Given the complex, multiple pathogeneses of acute liver inflammatory injury (ALIs), this study investigates BARI in a non-sterile murine model of fulminant hepatic failure to address existing management gaps.

**Methods:**

Mice were exposed to a toxic challenge with a single dose of lipopolysaccharide (LPS, 80 μg/kg, ip) and D-galactosamine (D-GaIN, 800 mg/kg, ip) on day 3. BARI (10 and 20 mg/kg, po) were administered prophylactically from day 1 to day 3 with the last dose of BARI given 2 h before LPS/D-GaIN.

**Results:**

Investigations revealed substantial lowering in liver function indices in BARI-treated groups after the pathogenic elevation in the LPS/D-GaIN group. These results coincided with remarkable reduction of necrosis and an improvement of hepatic architecture identified by the H&E staining in the BARI-treated groups compared to the disrupted histological features with substantial infiltration of inflammatory cells. The inflammatory cells observed in the LPS/ D-GaIN group represent a major source of ROS that contributes to nitrosative stress via the respiratory burst (NO), peroxidation of the PUFA of the cell membrane (MDA), and consumption of the cellular antioxidant molecules (TAC). This oxidative condition was reversed in BARI groups together with reestablishment of the antioxidant axis of Nrf2/HO-1 showing a remarkable capacity to restore redox equilibrium. The observed ameliorative potential of BARI is probably attributed to inhibition of IL-6 transcription, as confirmed by the inhibited activation of the transcription factor NF-κB, with subsequent inhibition of JAK2/STAT3. While curbing apoptosis as evidenced by reduced levels of cleaved caspase-3, BARI presented its ability to preserve the level of PI3K/AKT/ mTOR signaling.

**Conclusion:**

These findings highlight the protective capacity of BARI and its value for repurpose in medical management of acute liver failure.

## 1 Introduction

The immune system acts as the body’s primary means of defense during illnesses and maintains the body in a state of homeostasis. In autoimmune hepatitis, dysregulation can lead to immune-mediated injury to autologous tissues, including hepatocytes (Czaja, 2006; Sebode et al., 2018). Acute liver failure (ALF), an imminent and sometimes lethal occurrence for those without preexisting liver disease, can be abruptly brought on by this condition (Stravitz et al., 2023; Bernal et al., 2015).

Presently, the two primary therapies used for ALF are 1) liver transplantation, which is expensive but life-saving, and 2) N-acetylcysteine (NAC), that performs well for acetaminophen-induced ALF; nevertheless is not very helpful for other causes, such as viral or endotoxin-mediated liver damage (Tujios et al., 2022; Fontana and Bari, 2017; Ostapowicz et al., 2002) In order to manage non-acetaminophen ALF, there is an additional need for more protective options.

The global mortality rate for ALF remains elevated, with many deaths occurring in rural areas, despite advancements in medical technology and increased funding for critical care facilities (Stravitz et al., 2023; Bernal et al., 2015; Ostapowicz et al., 2002). These outcomes are brought on by a lack of suitable protective agents, rising costs, and limited access to specialized care.

Exploring therapies that can restore liver function before it entirely fails is one of the primary objectives of hepatology research. One promising candidate for pharmaceutical repurposing is baricitinib (BARI), a JAK1/2 inhibitor with wide availability and an established safety record. BARI is approved for rheumatoid arthritis and later employed in hyperinflammatory conditions involving COVID-19 (Stebbing et al., 2021; Stebbing et al., 2020). The JAK/STAT and NF-κB pathways, which are the main causes of cytokine storms, are impeded by this medication. By activating the Nrf2 and PI3K signaling pathways, the substance displays cytoprotective and antioxidant effects (Chen et al., 2024; Elbaset et al., 2024; Wu et al., 2022; Manning and Toker, 2017).

ALF-related conditions are increasingly reported in Middle Eastern countries, particularly in Saudi Arabia and Egypt. Recent assessments indicate a rising burden of liver diseases and noticeable shifts in etiological patterns across the Arab world (Almansoury et al., 2023). This pattern is consistent with broader findings showing increased contributions from drug-induced and immune-mediated liver injury and aligns with reports describing a growing hepatic disease burden in Middle Eastern populations (Alqahtani and Schattenberg, 2020).

According to some studies, BARI may reduce acute liver injury by simultaneously inhibiting inflammatory signaling pathways such as JAK2/STAT3 (Makabe et al., 2024; Gong et al., 2017; Xiong et al., 2021) and NF-κB (Gao et al., 2022; Lawrence, 2009; Liu et al., 2017), while promoting prosurvival/regenerative pathways (PI3K/Akt/mTOR) (Chen et al., 2024; Elbaset et al., 2024; Saxton and Sabatini, 2017). These mechanisms highlight its potential value as a repurposed in the therapy management of acute liver failure.

## 2 Materials and methods

### 2.1 Experimental animal groups

Male Albino mice weighing 25–35 g were acquired at the VACSERA: The Egyptian Organization of Biological Products and Vaccines, Giza, Egypt. During the experiments period, animals were kept in a controlled environment with a constant temperature of 25 °C ± 2 °C, 12 h of light/dark, and free access to food and water. All the experiments were conducted as per ethical principles accepted and approved by the Mansoura University Animal Care and Use Committee Approval No. MU-ACUC (PHARM.MS.25.02.132). Before samples, mice were anesthetized with secobarbital sodium, and all attempts were made to minimize distress and pain in handling and in sacrificing.

### 2.2 Drugs and chemicals

Lipopolysaccharide (LPS) *Escherichia coli* O111:B4 (Sigma-Aldrich, United States; catalog number L2630-10 mg) were dissolved in endotoxin-free distilled water and D-(+)-Galactosamine hydrochloride (D-GaIN) (≥99%, HPLC grade, Sigma-Aldrich, United States; catalog number G0500-5 mg was solubilized normal saline).

BARI was purchased as Baritava® tablets, containing 4 mg BARI per tablet by Hikma Pharmaceuticals (Sixth of October City, Giza, Egypt). Tablets were uniformly suspended in 0.5 g% carboxymethylcellulose (CMC) in the sterile distilled water to bring out final concentrations of 10 mg/10 mL and 20 mg/10 mL to be used orally (mice were administered 10 mL/kg).

The vehicle control was carboxymethylcellulose (CMC, 0.5 g%), which was acquired from Fisher Chemical, Leicestershire, United Kingdom.

### 2.3 Experimental study design

Thirty-two male mice were randomly assorted into four groups (n = 8 per group).

**Group I** – Normal Control: Received 0.5 g% CMC (10 mL/kg, orally) once daily for 3 days. On day 3, 1 h after the last dose, mice were injected intraperitoneally with normal saline (1 mL/kg).
**Group II** – LPS/D-GaIN Control: Received a single intraperitoneal injection of LPS/D-GaIN (80 μg/kg/800 mg/kg) (Tiegs et al., 1989; Dodson et al., 2019; Loboda et al., 2016).
**Group III** – BARI (10 mg/kg) (Matsushita et al., 2023) + LPS/D-GaIN: Received baricitinib (10 mg/kg, orally) once daily for 3 days. On day 3, 1 h after the last dose, mice were injected with LPS/D-GaIN (80 μg/kg/800 mg/kg).
**Group IV** – BARI (20 mg/kg) (Li et al., 2024) + LPS/D-GaIN: Received baricitinib (20 mg/kg, orally) once daily for 3 days. On day 3, 1 h after the last dose, mice were injected with LPS/D-GaIN (80 μg/kg/800 mg/kg). LPS/D-GalN dosage was determined based on previously published studies (El-Agamy et al., 2018). Oral doses of baricitinib (10–20 mg/kg) administered once daily for three consecutive days were selected according to prior experimental reports.

All animals were closely monitored during the experiment for any behavioral or physiological changes. Specific parameters observed included changes in body weight, signs of pain and distress like abnormal gait and hunched back, and changes in food and water intake as well. This design sought to assess the dose-dependent impacts of BARI on liver inflammation and damage.

### 2.4 Sample collection

Six hours after LPS/D-GaIN injection, blood samples were obtained following anesthesia by incising the conjunctival membranes and rupturing the ophthalmic venous plexus. Serum was separated by centrifugation at 3000 *g* for 15 min and stored at −20 °C for biochemical experiments. Liver tissues were rinsed with ice-cold saline and divided into two portions: one snap-frozen in liquid nitrogen for molecular characterization, and the other fixed in 10% buffered-formalin for histological and immunohistochemical evaluation.

### 2.5 Histopathological analysis

Formalin-fixed liver specimens were dehydrated, embedded in the paraffin, and thinly sectioned at 4–5 µm thickness. These sections were stained with hematoxylin and eosin (H&E) dyes and examined under the light microscope (Olympus, Japan) to assess necrosis, inflammation, and overall hepatic architecture using a semi-quantitative scale (0–4) and an average neutrophil count was determined for each group via counting four fields with the highest aggregates of polymorphonuclear leukocytes with segmented nuclei (Elshal and Hazem, 2022). Images were captured using a ToupCam digital camera (XCAM1080PHA; 2.8 pixels, United Kingdom) attached to an Olympus® microscope (CX23LEDRF; Japan).

### 2.6 Assessment of liver enzyme activities

Serum levels of LDH (Swemedbio, India), AST, and ALT (AGAPPE Diagnostics, India) were measured using the commercial kits according to the manufacturers’ protocols. Enzyme activities were expressed in U/L and used as indicators of hepatic cellular integrity and damage.

### 2.7 Estimation of oxidative stress and antioxidant parameters

Hepatic levels of MDA and NO were determined using commercial assay kits (Biodiagnostic Co., Cairo, Egypt). TAC was measured calorimetrically using the related Biodiagnostic kit, following the manufacturer’s directions.

### 2.8 Immunohistochemical evaluation of antioxidant transcription factors (Nrf2 and HO-1)

Immunohistochemical detection of Nrf2 and HO-1 was performed on paraffin-embedded liver sections to assess the hepatic antioxidant response. Primary antibodies for Nrf2 (Cat. No. MBS9217749, MyBioSource©, San Diego, CA, United States) and HO-1 (Cat. No. MBS9607909, MyBioSource©, San Diego, CA, United States) were used, followed by appropriate HRP-conjugated secondary antibodies (Cat. No. MBS705551, MyBioSource©, San Diego, CA, United States) that was visualized using 3,3′ -diaminobenzidine-peroxidase substrate and quantified by ImageJ software (United States). These transcription factors are key regulators of cellular defense and redox homeostasis. Images were captured using a ToupCam digital camera (XCAM1080PHA; 2.8 pixels, United Kingdom) attached to an Olympus® microscope (CX23LEDRF; Japan).

### 2.9 ELISA quantification of JAK2, STAT3, and IL-6 signaling components

Levels of phosphorylated JAK2 (p-JAK2 catalog NO. MBS7269976, MyBioSource©, San Diego, CA, United States), phosphorylated STAT3 (Y705 catalog NO. MBS1608617, MyBioSource©, San Diego, CA, United States), and IL-6 (catalog NO. MBS824703, MyBioSource©, San Diego, CA, United States), in liver homogenates were quantified by commercial kits of enzyme-linked immunosorbent assay (ELISA), Adhering to the guidelines established by the manufacturer. In summary, 100 µL of each sample or standard was added to wells pre-coated with specific capture antibodies and incubated at 37 °C for 2 hours. Following washing with phosphate-buffered saline supplemented with 0.05% Tween-20 (PBST), biotinylated detection antibodies were introduced, succeeded by the application of horseradish peroxidase (HRP)-conjugated streptavidin. The color development method commenced with tetramethylbenzidine (TMB) substrate and concluded with 2 N sulfuric acid. A microplate reader (BioTek Synergy HTX, United States) was employed to measure absorbance at 450 nm. We employed standard curves derived from known quantities of each analyte to determine the oncentrations, which were subsequently reported in pg/mL. In addition, Protein concentrations were estimated in liver lysates using bicinchoninic acid (BCA) protein colorimetric assay kit (G-Biosciences, United States).

### 2.10 ELISA determination of PI3K/Akt/mTOR signaling pathway

ELISA kits were used to quantify hepatic levels of PI3K (Cat. No. MBS162296, MyBioSource©, San Diego, CA, United States), Akt (Cat. No. ELK0791, ELK Biotechnology©, Denver, CO, United States), and mTOR (Cat. No. ELK8644, ELK Biotechnology©, Denver, CO, United States).

### 2.11 Immunohistochemical analysis of NF-κB and cleaved Caspase-3

Immunohistochemical staining was performed to detect NF-κB (p65 subunit, Cat. No. MBS669206, MyBioSource©, San Diego, CA, United States), and cleaved caspase-3 (Cat. No. E-EL-M0201, Elabscience©, Houston, Texas, United States) in liver sections. Visualization under light microscopy enabled morphological assessment of inflammation, apoptosis, and treatment response. Images were captured using a ToupCam digital camera (XCAM1080PHA; 2.8 pixels, United Kingdom) attached to an Olympus® microscope (CX23LEDRF; Japan).

### 2.12 Statistical analysis

GraphPad Prism V10 (GraphPad Software Inc, CA, United States) was operated for statistical analyses and graph construction. All data were denoted as mean ± standard error (SE) (Mean ± SE, n = 6-8). One-way ANOVA and *post hoc* Tukey‘s test were carried out as a statistical measure and in cases of non-parametric data, the analysis implied Kruskal Wallis test and Dunn’s multiple comparison by *post hoc* test. The symbols *, #, and @ indicated statistically significant differences compared to the normal control group, LPS/D-GaIN group, and BARI 10 mg/kg + LPS/D-GaIN group, respectively, at P < 0.05.

## 3 Results

### 3.1 Influence of baricitinib (BARI) on lipopolysaccharide/d-galactosamine (LPS/D-GaIN) induced changes in histopathological assessment of hepatic architecture

**FIGURE 1:**
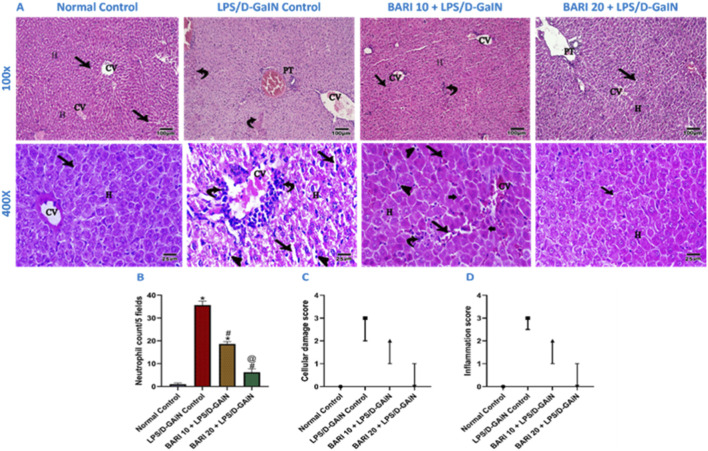
Representative photomicrographs of H&E-stained liver tissues demonstrating the protective effects of baricitinib against hepatic damage generated by LPS/D-GaIN. **(A)** Representative images of histopathology slides of liver tissues of different groups, **(B)** Neutrophil count/4 fields, **(C)** Cellular damage score. **(D)** Inflammation score. Normal control negative group showing normal liver architecture with polyhedral hepatocytes (H) arranged in strands with alternating blood sinusoids (arrows) forming a network around central vein (CV). Cells have slightly basophilic cytoplasm and vesicular rounded central nuclei. LPS/D-GaIN control group showed central vein congestion (CV), portal vein congestion in the portal tract (PT), sinusoidal dilation (arrows), increase Kupffer cells activity (arrow heads), cellular damage and inflammatory cells infiltration (curved arrows). BARI 10 mg + LPS/D-Gain treated group showed moderate central vein congestion (CV), moderate sinusoidal dilatation (arrows), inflammatory cells infiltration (curved arrows) and minimal Kupffer cells activity (arrow heads) among hepatocytes (H). Some hepatocytes are shrunken with dark acidophilic cytoplasm (thick arrows). BARI 20 mg + LPS/D-Gain treated group showing normal central vein (CV), separated by blood sinusoids (arrow), with no inflammatory cells infiltration among hepatocytes (H). Data are median ± interquartile ranges (n = 4), Kruskal Wallis test followed by *post hoc* Dunn’s multiple comparison test were used to compare between groups. *, #, and @ represent the levels of statistical significance compared to the Normal control, LPS/D-GaIN control groups, and 10 mg/kg dose respectively. Low magnification is denoted by X: 100 bar 100 μm. and high magnification shows X: 400, while bar represents 25 µm.

The histopathological examination of liver sections stained with dyes of hematoxylin and eosin demonstrated morphological condition of hepatic tissues. It was observed that the livers tissues from the LPS/D-GaIN treated group of mice contain profound hemorrhagic necrosis, degeneration of hepatic lobules, and substantial infiltration by inflammatory cells (Figures 1A–D). In contrast, the mice groups co-administered with BARI demonstrated normal structural integrity and preservation (Figures 1A–D). Mice administered with lower doses of 10 mg/kg of BARI exhibited significant protection, characterized by reduced necrosis and inflammatory infiltrates. However, it was noteworthy that the higher doses of 20 mg/kg had significantly (p < 0.05) more protection compared to doses of 10 mg/kg, nearly reinstating normal liver architecture with minimal tissue damage or inflammation. Hence, our results show that BARI protects liver tissues in dose-dependent manner.

### 3.2 Influence of baricitinib (BARI) on lipopolysaccharide/d-galactosamine (LPS/D-GaIN) induced changes in liver function biomarkers (AST, ALT, and LDH)

**FIGURE 2:**
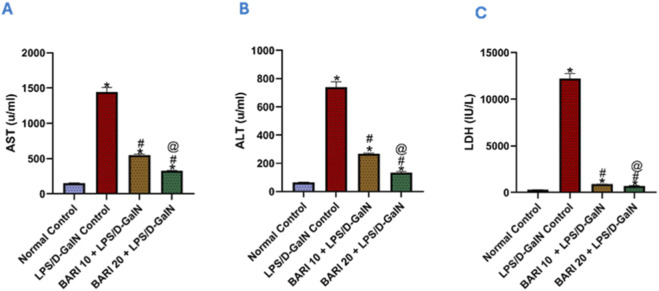
Influence of baricitinib (BARI) on lipopolysaccharide/d-galactosamine (LPS/D-GaIN) induced changes in liver function biomarkers. **(A)** Serum alanine aminotransferase (ALT), **(B)** Serum aspartate aminotransferase (AST), and **(C)** Serum lactate dehydrogenase (LDH). Data are mean ± standard error (n = 8), One-way ANOVA was followed by *post hoc* Tukey-Kramer multiple comparisons to compare mean values. *, # and @ represent levels of statistical significance compared to the normal control, LPS/D-GaIN control and BARI 10 + LPS/D-GaIN groups, respectively.

In order to elucidate the protective effects of BARI on liver, the LPS/D-GaIN was administered to a group of mice resulting in significant liver damage, elevating AST by more than six times, ALT by almost ten times, and LDH by approximately fifteen times compared to normal control group (Figures 2A–C). The administration of BARI on incremental doses significantly (p = 0.05) reduced these surges in a dose-dependent way (Figures 2A–C). Furthermore, the 10 mg/kg dosage reduced AST by almost 50%, ALT by over 50%, and LDH by more than 70% compared to LPS/D-GaIN group. Moreover, at dosage of 20 mg/kg, AST and ALT levels returned to nearly normal control group, while LDH levels decreased by more than 90% relative to the LPS/D-GaIN group of mice, indicating effective protection of hepatocytes. These results represent the marked effectiveness of BARI to prevent hepatotoxicity against offending agents.

### 3.3 Influence of baricitinib (BARI) on lipopolysaccharide/d-galactosamine (LPS/D-GaIN) induced changes in cellular oxidant profile (TAC, MDA, and NO)

**FIGURE 3:**
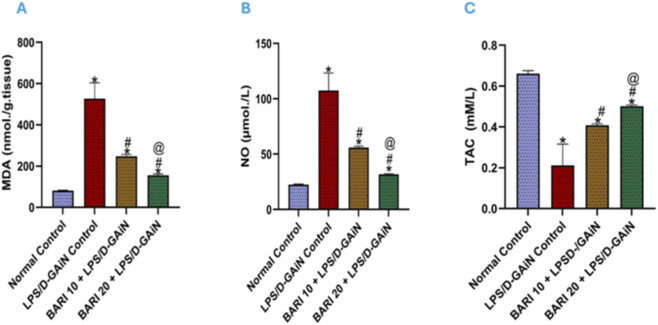
Influence of baricitinib (BARI) on lipopolysaccharide/d-galactosamine (LPS/D-GaIN) induced changes in cellular oxidant profile. **(A)** Malondialdehyde (MDA), **(B)** Nitric oxide (NO), and **(C)** Total antioxidant capacity (TAC). Data represent mean ± standard error (n = 8), One-way ANOVA was followed by application of *post hoc* Tukey-Kramer multiple comparisons to compare mean values. *, # and @ represent statistical significance compared to the normal control, LPS/D-GaIN control and BARI 10 + LPS/D-GaIN groups, respectively.

After finding out the hepatoprotective activities of BARI, we decided to explore the underlying mechanisms of protective processes. It was observed that LPS/D-GaIN treatment exerted a substantial oxidative burden, marked by elevated MDA and NO levels while extensively alleviating TAC in comparison to the normal control group of animals (Figures 3A–C). The application of BARI corrected this imbalance in a dose-dependent fashion: the 10 mg/kg dosage reduced MDA and NO levels by about 50% and increased TAC by approximately 73%, whereas the 20 mg/kg dosage further decreased MDA and NO by about 70% and restored TAC by approximately 79% (Figures 3A,B). BARI 20 mg/kg demonstrated significant antioxidant capabilities by increasing TAC status and significantly diminished oxidative stress markers. Hence, our results manifest that BARI positively regulates the oxidative pathways leading to hepatoprotective actions.

### 3.4 Influence of baricitinib (BARI) on lipopolysaccharide/d-galactosamine (LPS/D-GaIN) induced changes in antioxidant transcription pathways (NRF2 and HO-1)

**FIGURE 4:**
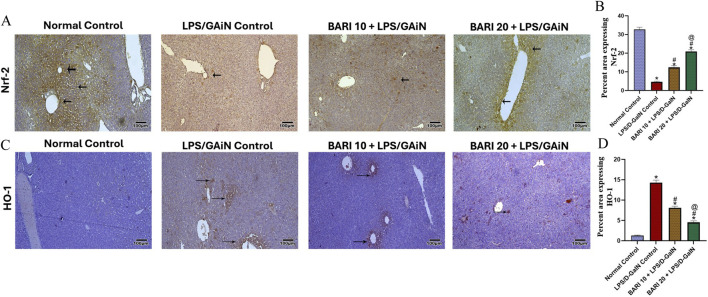
Representative photomicrographs of IHC-stained liver tissues demonstrating the expressions of Nrf2 and HO-1. **(A)** Photomicrographs of mice liver tissues sections of Normal control group displaying excess expression of Nrf2 immunostaining (arrows). LPS/D-GaIN control group showed weak Nrf2 immunostaining (arrow). BARI 10 mg + LPS/D-GaIN treated group showed moderate Nrf2 immunostaining (arrow). BARI 20 mg + LPS/D-GaIN treated group showing excess Nrf2 immunostaining (arrows). **(B)** Area of Nrf2 expression (%) **(C)** Photomicrographs of mice liver tissues sections of Normal control group showing negative HO-1 immunostaining. LPS/D-GaIN control group showed marked HO-1 immunostaining (arrows). BARI 10 mg + LPS/D-GaIN treated group showed moderate HO-1 immunostaining (arrows). BARI 20 mg + LPS/D-GaIN treated group showing minimal HO-1 immunostaining (arrow). and **(D)** Area of HO-1expression (%). Data show means ± standard error (n = 4), One-way ANOVA followed by multiple comparisons using *post hoc* Tukey-Kramer to compare mean values. *, # and @ represent statistical significance compared to the normal control, LPS/D-GaIN control and BARI 10 mg + LPS/D-GaIN groups, respectively. The scale bar represents 50 µm.

To delineate the mechanism of oxidative control, we performed IHC of Nrf2, which is documented as master transcriptional factor of antioxidant mechanisms. In the LPS/D-GaIN treated group, hepatic Nrf2 expression was profoundly reduced (Figures 4A,B), resulting in the compensatory elevation of HO-1 levels representing the maladaptive stress response (Figures 4C,D). Conversely, BARI administration reinstated typical antioxidant signaling status: at 10 mg/kg, Nrf2 expression significantly elevated, and HO-1 normalized; at 20 mg/kg, Nrf2 expression further increased, and HO-1 levels aligned with those of the healthy control group, indicating restored redox homeostasis (Figures 4A–D). Thus, our IHC results depict that BARI induces antioxidant benefits at transcription level through Nrf2 elevation and HO-1 repression.

### 3.5 Influence of baricitinib (BARI) on lipopolysaccharide/d-galactosamine (LPS/D-GaIN) induced changes in JAK2/STAT3/IL-6 axis

**FIGURE 5:**
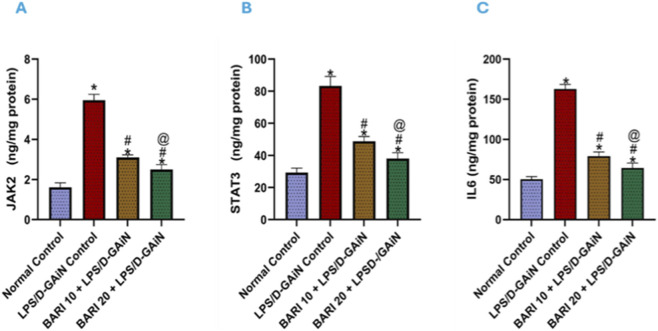
Influence of baricitinib (BARI) on lipopolysaccharide/d-galactosamine (LPS/D-GaIN) induced changes in the expression of **(A)** Janus kinase 2 (JAK2), **(B)** signal transducer and activator of transcription 3 (STAT3), and **(C)** Interleukin-6 (IL-6). Data are mean values ± standard error (n = 4), One-way ANOVA for group comparisons followed Tukey-Kramer multiple comparisons to compare mean values. *, # and @ represent levels of statistical significance compared to the normal control, LPS/D-GaIN control and BARI 10 + LPS/D-GaIN groups, respectively.

Following observation of oxidative stress reduction by BARI, we were impelled to explore the implication of inflammatory mediators and a related pathway. Hence, we examined the level of IL-6 in our designed groups. It was noted that the exposure to LPS/D-GaIN resulted in significant (p < 0.05) induction of IL-6 levels and a marked elevation in hepatic JAK2 and STAT3 activation relative to the control normal group (Figures 5A–C). Meanwhile, the administration of BARI significantly diminished this activation in a dose-dependent fashion. At a dosage of 10 mg/kg, JAK2 (Figure 5A) and STAT3 (Figure 5B) levels diminished by around 45%, while IL-6 (Figure 5C) levels declined by nearly 50%. At a dosage of 20 mg/kg, these decreases were further diminished to approximated 55%–60%, indicating that the pro-inflammatory JAK/STAT axis accompanied by IL-6 expression were significantly downregulated. Altogether, BARI effectively suppresses JAK2/STAT3/IL-6 axis.

### 3.6 Influence of baricitinib (BARI) on lipopolysaccharide/d-galactosamine (LPS/D-GaIN) induced changes in PI3K/AKT/mTOR inflammatory pathway

**FIGURE 6:**
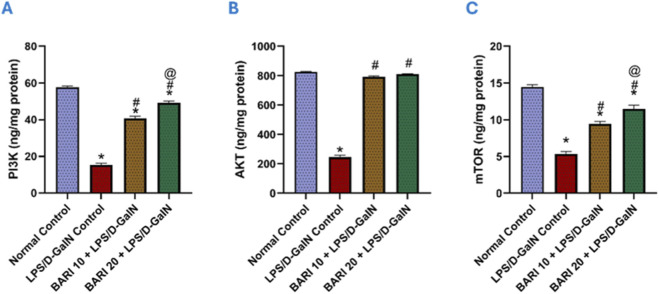
Influence of baricitinib (BARI) on lipopolysaccharide/d-galactosamine (LPS/D-GaIN) induced changes in the expression of **(A)** phosphatidyl inositol-3-kinase (PI3K), **(B)** protein kinase B (AKT), and **(C)** mammalian target of rapamycin (mTOR). Data are mean values ± standard error (n = 4), One-way ANOVA for group comparison followed Tukey-Kramer multiple comparisons to compare mean values. *, # and @ represent statistical significance compared to the normal control and LPS/D-GaIN control and BARI 10+ LPS/D-GaIN groups, respectively.

To further reveal the regulation of inflammatory pathways by BARI, we investigated the expression of PI3K/Akt/mTOR signaling. The PI3K/Akt/mTOR signaling cascade was significantly repressed in LPS/D-GaIN group of animals, as seen by the reduced levels of PI3K (∼74%) (Figure 6A), Akt (∼70%) (Figure 6B), and mTOR (∼63%) (Figure 6C) relative to the normal control. BARI reinstated this pathway in a dose-dependent fashion: 10 mg/kg elevated PI3K to approximately 63%, Akt to approximately 69%, and mTOR to approximately 43% above the disease cohort challenged with LPS/D-GaIN, whereas 20 mg/kg boosted these levels to approximately 69%, approximately 70%, and approximately 53%, respectively, signifying augmented survival signaling and enhanced cellular recuperation (Figures 6A–C).

### 3.7 Influence of baricitinib (BARI) on lipopolysaccharide/d-galactosamine (LPS/D-GaIN) induced changes in the expression of the transcription factor NF-κB and the apoptotic marker caspase-3

**FIGURE 7:**
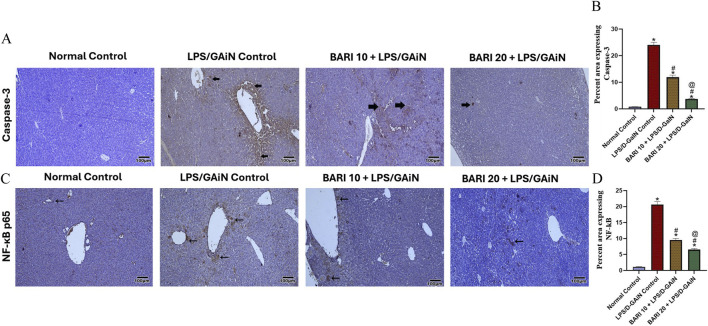
Representative photomicrographs of IHC-stained liver tissues demonstrating the expressions of NF-κB and caspase-3. **(A)** Photomicrographs of mice liver tissues sections of normal control group showing negative caspase 3 immunostaining. LPS/D-GaIN control group showed marked caspase 3 immunostaining (thick arrows). BARI 10 + LPS/D-GaIN treated group showed moderate caspase 3 immunostaining (thick arrow s). BARI 20 + LPS/D-GaIN treatment group showing minimal caspase 3 immunostaining (thick arrow). **(B)** Area of cleaved caspase-3 expression (%), **(C)** Photomicrographs of mice liver tissues sections of normal control group showing mild NF-κB immunostaining (arrow). LPS/D-GaIN control group showed marked NF κB immunostaining (arrows). BARI 10 + LPS/D-GaIN treated group showed moderate NF-κB immunostaining (arrows). BARI 20 + LPS/D-GaIN treated group showing minimal NF-κB immunostaining (arrow). and **(D)** Area of NF-κB expression (%). Data are mean values ± standard error (n = 4), One-way ANOVA to compare different groups followed Tukey-Kramer multiple comparisons to compare mean values. *, # and @ represent statistical significance compared to the normal control, LPS/D-GaIN control and BARI 10 + LPS/D-GaIN groups, respectively. The scale bar represents 50 µm.

Finally, we ought to confirm the anti-inflammatory and survival roles of BARI by determining the expressions of transcription factor NF-κB and apoptotic marker caspase-3. The LPS/D-GaIN-induced disease model elicited substantial expression of cleaved caspase-3 (Figures 7A,B) and upregulation of hepatic NF-κB (Figures 7C,D), indicating severe inflammation reactions and induction of apoptotic cell death. BARI 10 mg/kg pretreatment significantly reduced NF-κB levels by roughly 93% (Figures 7C,D) and caspase-3 levels by approximately 64% (Figures 7A,B). The 20 mg/kg dosage exhibited greater efficacy compared to lower doses of 10 mg/kg, reducing NF-κB by around 94% and caspase-3 by roughly 82%. The concentrations of caspase-3 diminished from around 25 ng/mg protein in diseased animals to roughly 10 ng/mg at dosage of 10 mg/kg and 4 ng/mg at 20 mg/kg. The results indicate that BARI possesses anti-inflammatory and anti-apoptotic properties via transcriptional deactivation of NF-κB and downregulation of apoptotic marker caspase-3.

## 4 Discussion

This study has manifested the role of BARI in hepatic protection against harmful stimuli. Hence, understanding the underlying mechanisms of hepatic injury and its pharmacological modulation is essential for developing effective protective strategies. The investigation of the present study highlights the experimental observations in light of previous research findings, aiming to clarify the mechanistic role of BARI in hepatoprotective actions. The findings of experimental data are evaluated from both molecular and functional perspectives to elucidate the connections between oxidative stress, inflammatory signaling, and cell survival. This work demonstrates defensive mechanisms of BARI against LPS/D-GaIN based liver damage and providing molecular insights in this process.

### 4.1 Histopathological assessment of liver architecture (H&E staining)

BARI, acting as a selective JAK1/JAK2 inhibitor, exerts its substantial protective activities on the hepatic architecture against LPS/D-GaIN-induced acute injury. The structural preservation of lobular organization and reduction in necrotic foci has been observed in this work, which is in line with previous histopathological findings by (Liu et al., 2017; Matsushita et al., 2023). It has been reported in those studies that inhibition of JAK-pathway mitigates inflammatory-mediated cellular disruption. The near-normal structure in the 20 mg/kg group implies the efficient suppression of necrotic and inflammatory changes and enhanced hepatic regenerative capabilities. This cell architectural preservation corroborates the biochemical recovery noted in subsequent parameters, collectively reinforcing baricitinib’s role in maintaining tissue integrity through immunomodulatory control.

### 4.2 Baricitinib effects on biomarkers of liver functions (AST, ALT, and LDH)

The strong hepatoprotective potential of BARI is proven by the dose-dependent reductions in AST, ALT, and LDH levels. These enzymes act as significant indicators of hepatic cytolysis and are usually produced following hepatocyte membrane rupture. Their decline following BARI treatment shows stability of hepatocyte membranes and restored metabolic equilibrium (Chen et al., 2024; Samuel et al., 2023). reported similar decreases in the same biomarkers, attributing enzyme normalization to JAK/STAT inhibition and suppression of cytokine-driven degradation. Therefore, the current data support the notion that BARI prevents downstream inflammatory amplification by restricting IL-6/JAK2/STAT3 signaling cascades, which in turn mitigates hepatocellular porosity.

### 4.3 Baricitinib effects on oxidative stress biomarkers (MDA, NO, and TAC)

The primary component of LPS/D-GaIN-mediated hepatotoxicity is oxidative stress. Its strong antioxidant capacity is demonstrated by the reductions in MDA and NO as well as the recovery of TAC after BARI intervention. These improvements are consistent with the oxidative normalizations documented by (Li et al., 2024; Samuel et al., 2023; Wu et al., 2022; Makabe et al., 2024), who underscored that JAK inhibition reduces cytokine storm activity, so indirectly restricting reactive oxygen species. The current results further indicate that BARI stimulates endogenous antioxidant responses, potentially by lowering the synthesis of nitric oxide produced from iNOS and secondarily activating Nrf2 (Ma, 2013; Villanueva-Paz et al., 2021; Loboda et al., 2016). The cumulative significance of JAK/STAT–redox cross-talk in regaining hepatic biochemical stability is shown by the dose-dependent pattern identified (Makabe et al., 2024; Stebbing et al., 2020).

### 4.4 Effects of baricitinib on immunohistochemistry levels of antioxidant transcription pathways involving NRF2 and HO-1

The effective reactivation of the endogenous antioxidative defense mechanism is demonstrated by HO-1 levels returning to baseline and restoration of Nrf2 expression in livers tissues of BARI-treated mice. Nrf2 downregulation impairs the transcription of antioxidant response elements under LPS/D-GaIN stress, yet compensatory hyperexpression of HO-1 suggests cellular stress. In line with (Elbaset et al., 2024; Wu et al., 2022), who found that JAK inhibition indirectly disrupts the Nrf2/HO-1 axis through reduction of inflammatory stress, BARI treatment restored the regulatory balance between these factors. These findings imply that the hepatoprotection provided by BARI include molecular improvements in detoxification and redox-responsive gene expression in addition to cytokine suppression.

### 4.5 Baricitinib effects on JAK2/STAT3/IL-6 signaling

The current study clearly illustrates that in the LPS/D-GaIN mice model, BARI interferes with the JAK2/STAT3/IL-6 signaling circuit that promotes inflammatory and apoptotic responses. The observed drop in IL-6 and reduction of JAK2 and STAT3 activation are consistent with mechanistic findings by (Chen et al., 2024; Liu et al., 2017). BARI minimizes hepatocyte mortality and alleviates the cytokine storm by preventing the phosphorylation of JAK2/STAT3, which suppresses the transcription of pro-inflammatory cytokines like TNF-α and IL-1β. The enzyme stabilization and morphological recovery documented in the result section are explained by this targeted regulation, indicating that molecular impairment of this cytokine axis is essential to the overall protective profile of BARI.

### 4.6 Effect of baricitinib on PIK3/AKT/mTOR survival signaling pathway

A cytoprotective mechanism associated with greater hepatocyte survival has been demonstrated by upregulation of PI3K/AKT/mTOR signaling demonstrated by mice treated with BARI. Under endotoxin-induced injury, this mechanism is usually impaired, resulting in autophagy disorder and apoptosis. The administration of BARI restores higher cell viability and metabolic resilience. The identical findings have been presented by (Li et al., 2024; Wang et al., 2021; Saxton and Sabatini, 2017; Xiong et al., 2021), who determined that hepatic recovery after JAK inhibition depended largely on PI3K/AKT/mTOR regulation. The current study’s correlation between reduced oxidative indices and PI3K/AKT reactivation illustrates a linked survival–redox response mediated by BARI. PI3K/AKT/mTOR signaling promotes cell cycle regulation and associated cell survival and cell growth. This signaling pathway has been impeded by treatment with LPS/D-GaIN.

### 4.7 Effect of baricitinib on immunohistochemistry expression of NF-KB and Caspase-3

The anti-inflammatory and anti-apoptotic effects of BARI have been confirmed by decreased expression of NF-κB and caspase-3 after administration of the drug. The observed downregulation originates from upstream inhibition of JAK/STAT-dependent inflammatory mediators, which effectively mitigates the NF-κB cascade though BARI does not directly inhibit NF-κB. According to (Lawrence, 2009; Liu et al., 2017; Gong et al., 2017), the concurrent reduction in caspase-3 suggests that the apoptotic signaling processes have been interrupted. Together, our results show the way blocking the IL-6/JAK/STAT3/NF-κB axis may interrupt the inflammatory cycle and prevent hepatocyte death, offering a coherent explanation for the biochemical and histological recovery witnessed throughout this investigation.

### 4.8 Mechanistic pathway integration

The integrative processes investigated in the present article show how BARI exerts its hepatoprotective advantages by concurrently regulating oxidative, inflammatory, and survival pathways. LPS/D-GaIN first stimulates the release of NF-κB-mediated cytokines, which intensifies the inflammatory cascade (Liu et al., 2017). By preventing JAK1/2 signaling, BARI interrupts the IL-6/JAK/STAT positive feedback loop that perpetuates inflammation (Chen et al., 2024). BARI contributes to restore metabolic equilibrium by suppressing NF-κB activation and oxidative mediators including MDA and NO as the process advances toward resolution (Samuel et al., 2023). Lastly, hepatocellular equilibrium restoration and cellular survival are facilitated by the activation of the Nrf2/HO-1 and PI3K/Akt/mTOR pathways (Elbaset et al., 2024; Saxton and Sabatini, 2017). This robust interaction illustrates the complex hepatoprotective effect of BARI and is consistent with findings from previous experimental trials (Matsushita et al., 2023).

### 4.9 Limitations and future directions

Future research ought to investigate chronic inflammatory and fibrotic paradigms, appropriate dosage schedules, and metabolic end-points, though the data related to BARI indicated a strong hepatoprotective potential in the acute phase. There is need to extend and monitor combinatory therapy of BARI with antioxidants or anti-fibrotic drugs that could enhance treatment effectiveness considerably. In addition, to support the conclusions of the current study, complementary western blot analyses of the proteins under investigation may be considered. Moreover, in order to confirm the translational safety and usefulness of BARI in immune-mediated hepatic failure, the clinical trials are still necessary (Li et al., 2024; Samuel et al., 2023).

## 5 Conclusion

BARI is an appealing choice as a protective agent in ALF because of its proven safety profile in autoimmune and inflammatory diseases as well as its prior use in inflammatory disorders. The long-term implications of BARI in models of chronic liver disease, the controlling mechanisms of undetected variations, and translation studies in a medical facility need to be explored further in future study to build on these findings. All of these results support BARI’s potential as a next-generation anti-inflammatory treatment for hepatology. Future preclinical and clinical studies involving various approaches should assess the safety and efficacy features in individual randomized trials.

## Data Availability

The original contributions presented in the study are included in the article/supplementary material, further inquiries can be directed to the corresponding author.
